# Snare-Assisted Retrieval of an Embolized Long Coronary Guidewire From the Aortic Arch

**DOI:** 10.7759/cureus.59707

**Published:** 2024-05-05

**Authors:** Dibyasundar Mahanta, Kowtarapu Sai Karthik, Saran Mohanan, Satyapriya Mohanty, Debasish Das

**Affiliations:** 1 Cardiology, SUM Hospital, Bhubaneswar, IND; 2 Cardiology, All India Institute of Medical Sciences, Bhubaneswar, Bhubaneswar, IND; 3 Cardiothoracic Vascular Surgery, All India Institute of Medical Sciences, Bhubaneswar, Bhubaneswar, IND

**Keywords:** guidewire, coronary, embolized, retrieval, assisted, snare

## Abstract

We report an extremely rare case of gooseneck snare-assisted retrieval of an embolized coronary guidewire from the aortic arch in an elderly male scheduled for a transradial coronary angiogram for unstable angina. In this case, the proximal end of the embolized coronary guidewire could not be retrieved from the brachial artery nor the roomy aortic root using a flower petal snare. The key takeaway from this case is that an embolized coronary guidewire can be successfully retrieved with a gooseneck snare from its proximal end in a moderately spacious area like the aortic arch.

## Introduction

A broken and embolized coronary guidewire is a critical challenge in the cardiac catheterization laboratory. Inside the coronary artery, an embolized coronary guidewire acts as a nidus for thrombosis, potentially leading to coronary thrombosis. When a coronary guidewire embolizes into the cerebral circulation, it poses a catastrophic risk, potentially resulting in cerebral thrombosis and ischemic stroke. Conversely, embolized coronary guidewires in the distal peripheral circulation may behave more benignly. Small guidewires embolized into the peripheral circulation are typically secured to the arterial wall with a bare-metal stent, whereas longer embolized guidewires usually require snare-assisted [1] or surgical retrieval. We report a case involving a broken coronary guidewire in the brachial artery that migrated into the aortic root due to guide catheter manipulation and was subsequently retrieved successfully with a gooseneck snare from the aortic arch. The distal broken part of the coronary guidewire could not be caught with a snare in the distal radial artery, as it was straight and slippery and the space in the distal radial artery was limited.

## Case presentation

**Figure 1:**
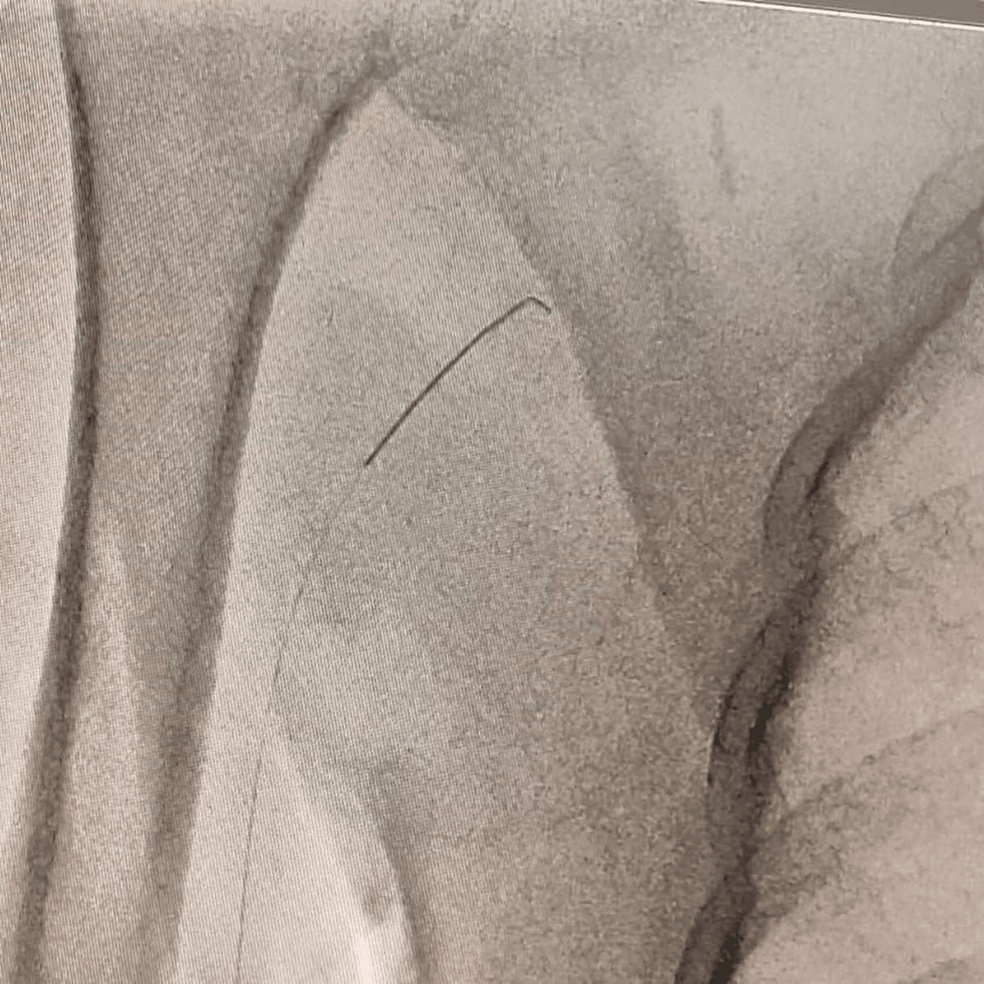
Broken coronary guide wire in brachial circulation

**Figure 2:**
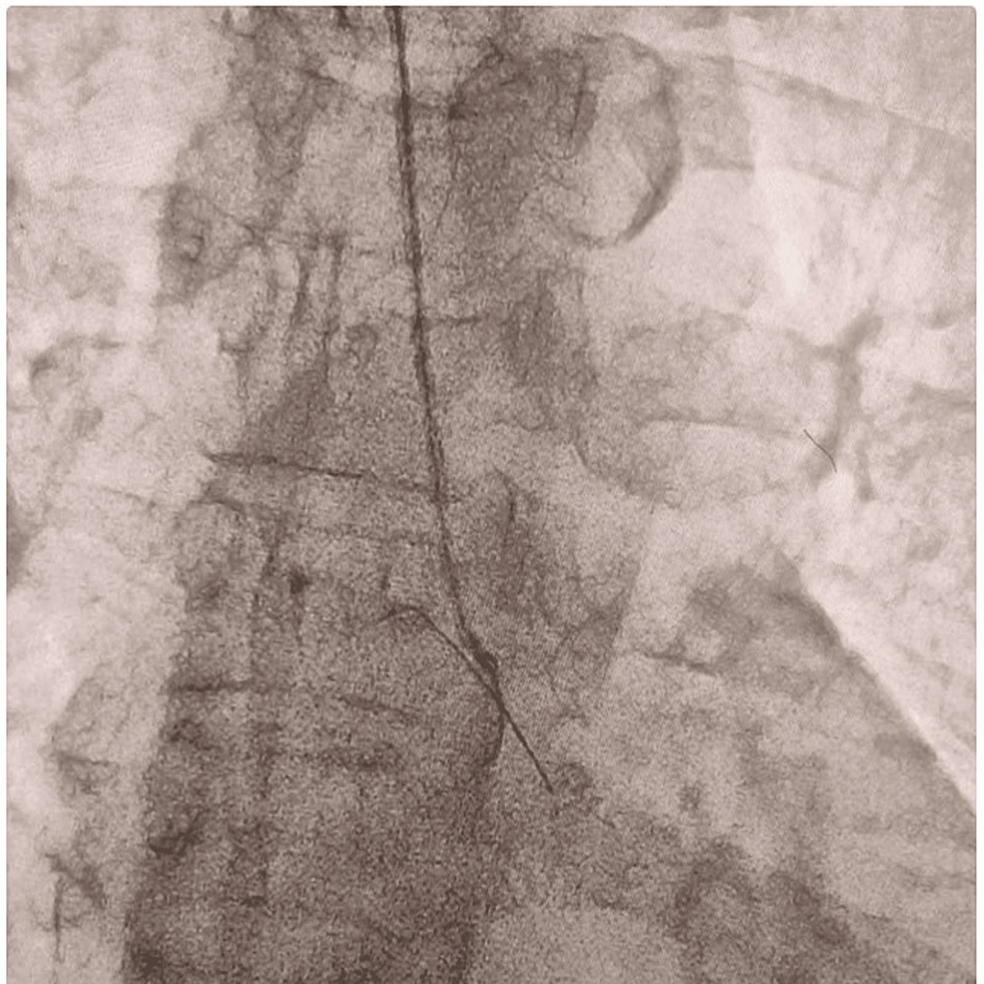
Snaring of the embolized coronary guide wire with Gooseneck snare from the aortic root

A 65-year-old male, who is diabetic and hypertensive, nonalcoholic, nonsmoker, presented to the cardiology outpatient department (OPD) with rest angina over the last three days, accompanied by diaphoresis and shortness of breath. He had a history of a transient ischemic attack with right hemiparesis two years earlier, from which he had fully recovered without any residual neurological deficits. During the clinical examination, his pulse rate was 88 beats per minute, and his blood pressure was 140/90 mm Hg in the right arm while in a supine position. The cardiovascular system examination revealed the presence of a left ventricular fourth heart sound (LV S4). His electrocardiogram (ECG) showed down-sloping ischemic ST depression with T wave inversion in the anterolateral leads. Echocardiography revealed no regional wall motion abnormalities with preserved left ventricular systolic function (EF=60%). He underwent a right transradial coronary angiogram for ongoing angina. The resident punctured the right radial artery with a Terumo puncture needle, but the blood jet was not robust. He could not advance the routine 0.035" Terumo wire through the plastic hub. Consequently, he switched to a 0.014'' coronary guidewire, which traversed well up to the brachial artery. While negotiating the sheath, the resident did not provide a sufficient nick, which required much harder force to push the 6F radial sheath inside. The 6F radial sheath was negotiated inside, but while pulling the coronary guidewire, the resident noted that only a small portion of the guidewire came out. Fluoroscopy revealed that the guidewire was broken at the distal end in the radial artery with the proximal end in the brachial artery (Figure 1). A 6F Judkins Right (JR) guide catheter was advanced into the distal part of the radial artery, and a flower petal snare was used in an attempt to catch the wire in its distal part, but it was unsuccessful as the distal part was straight and smooth. The decision was then made to catch the wire from the proximal part in the brachial artery with the same flower petal snare. However, while negotiating the guide catheter beside the wire, the wire migrated into the aortic root along with the guide catheter. The aortic root, dilated due to age, was quite roomy. Despite repeated attempts with the flower petal and gooseneck snares, the wire could not be caught in the dilated aortic root (Figure 2) due to its roomy nature. The decision was then made to pull back the guide catheter into the aortic arch; as the guide catheter was pulled back into the aortic arch, the guidewire also moved back into the aortic arch, where it was successfully caught with a gooseneck snare near the innominate artery junction. The snare-coronary guidewire assembly was retrieved through the 6F radial sheath. The entire radial sheath, snare, and guidewire assembly were then removed in toto. The patient developed a small radial hematoma, which was managed conservatively. A transradial angiogram from the left radial artery in the subsequent setting revealed critical triple vessel coronary artery disease. He was advised to undergo coronary artery bypass surgery and was doing well post-surgery in follow-up. Post snare-assisted retrieval of the coronary guidewire, the brachial artery showed multiple small dissections without any flow-limiting thrombosis and was managed with anticoagulants.

## Discussion

A broken coronary guidewire is a nightmare in the coronary intervention laboratory. Embolization of the coronary guidewire into the cerebral circulation is a catastrophe that results in thrombotic stroke. The crux of the present case was that it could not be snared with a flower petal snare from its distal end as it was straight and slippery in nature, and the space inside the distal radial artery was less roomy. Hence, it was decided to snare it from the proximal end, which was curved with more space in the aortic arch. The second interesting finding in this index case was that it could not be snared with a flower petal snare with its small snaring end, whereas a medium curve gooseneck snare was able to retrieve it from the aortic arch-innominate artery junction. It was difficult to snare the embolized guidewire from the aortic root as it was much roomier due to age-related unfolding. Small broken coronary guidewires are crushed in the circulation by putting a stent over them [2-4] or left as such in situ [5,6], whereas it is impossible to do the same with a long broken guidewire in the peripheral circulation. The most important complication following snaring is the development of bacterial sepsis due to the long procedure time required to retrieve a small guidewire from the roomy peripheral circulation. A different option in the present case would have been obtaining surgical access at the distal radial or brachial site and subsequent retrieval of the coronary guidewire, which may have increased the chance of post-procedure sepsis. The most important caution in the forearm embolization of a broken guidewire is that the forearm should be immobilized until it is snared out, as further embolization into the cerebral circulation is a catastrophe. An embolized coronary guidewire is usually crushed with a second stent in the coronary artery as it acts as a nidus for thrombus formation. Most of the cardiac catheterization laboratories use sterilized old guidewires which are more prone to kink and break during coronary intervention. Young interventional cardiologists should not apply inadvertent force over the coronary guidewire when it meets resistance; otherwise, it would result in an unwanted catastrophe in the coronary intervention laboratory.

## Conclusions

We report an extremely rare case of gooseneck snare-assisted retrieval of the embolized coronary guidewire from the aortic arch-innominate junction. The learning point in the present case was that it was snared at its curved proximal end in a medium roomy space like an aortic arch-innominate junction. Young interventional cardiologists should not apply undue force on the coronary guidewire when it meets resistance to avoid such nightmares in the cardiac catheterization laboratory.
